# Cultivating Healthy Connections: Exploring and Engineering the Microbial Flow That Shapes Microbiomes

**DOI:** 10.1128/mSystems.00863-21

**Published:** 2021-10-05

**Authors:** Travis J. Wiles

**Affiliations:** a Department of Molecular Biology & Biochemistry, University of California, Irvine, California, USA

**Keywords:** antibiotic resistance, beneficial microbes, host-microbe interactions, host-microbe systems biology, microbial ecology, microbial transmission, microbiome, microbiome engineering

## Abstract

Our view of the microbial world has undergone a radical transformation over the past decade. For most of the 20th century, medical microbiological research was focused on understanding the virulent nature of disease-causing pathogens. More recently, advances in DNA sequencing methodologies have exposed a wider diversity of microscopic wildlife that associate with our bodies and the environments around us, and the unexpected roles they play in supporting our health. Our expanding view of the microbial world is now motivating therapeutic interventions that are based not just on the elimination of nefarious pathogens but the nurturing of beneficial microbiomes. In this Commentary, I consider how our historically pathogen-based view of host-microbe interactions may be limiting the scope of new and alternative strategies for engineering microbiomes. I suggest that recognizing the therapeutic potential of the ongoing microbial transmission that connects microbiomes could illuminate unexplored opportunities for cultivating healthy host-microbe relationships.

## COMMENTARY

## OUR EXPANDING VIEW OF THE MICROBIAL WORLD

Over the last decade there has been a collective awakening across scientific disciplines and society to the multitudes of microorganisms that inhabit our bodies and the environments we occupy. Compelling discoveries of how indigenous microbes support our health and predispose us to disease are being made at a rapid clip. Our microbiome can facilitate the harvesting of nutrients and energy from our diet (1), protect us from pathogens (2), modulate our susceptibility to chronic and complex disorders like asthma (3) and autoimmune diabetes (4), and influence our likelihood of developing cancer (5) and whether we will respond to treatment (6). The mechanisms underlying our interactions with microbes and their potential to serve as therapeutic targets are starting to be clarified using experimental animal models such as mouse, zebrafish, and several invertebrate species (e.g., bobtail squid, fruit flies, and nematodes). It is incredible to think that obtaining a complete picture of human biology (and the biology of other animals) was likely never possible without considering the microscopic wildlife that surrounds us. Ultimately, the insights generated from modern microbiome research are catalyzing dramatic shifts in how we view and interact with the microbial world (7).

To appreciate how our relationship with the microbial world is changing, it is important to consider how it formed in the first place (7). The original microbial envoys were mostly pathogens (e.g., Vibrio cholerae, Bacillus anthracis, Yersinia pestis, and Mycobacterium tuberculosis), and since the widespread acceptance of the germ theory of disease over 120 years ago, pathogens have dominated our conceptual framework for understanding microbes. Pathogens decided the terms of our initial engagements and dictated the battlefront for our confrontations: our bodies. In response, a tremendous amount of research throughout the 20th century was devoted to deciphering the genes and pathways that infectious microbes use to invade and replicate within our tissues and cells, and how these encounters lead to acute and life-threatening afflictions. As a result, we have developed countermeasures such as broad-spectrum antibiotics and vaccines to rid our bodies of unwanted microbial intruders. Our historically adversarial relationship with microbes now stands in sharp contrast to new microbiome-based therapies that are focused on treating disease by cultivating microbes, not by eradicating them. But as we strive to learn how to grow healthy microbiomes, it is also worth considering how our conflicts with pathogens have shaped our assumptions about host-microbe interactions and their underlying mechanisms.

It can be argued that pathogens have imprinted on us the expectation that for any given disease or health-promoting property of the microbiome, there should be a single prominent microbe—at a concurrent place and time—that is responsible. Accordingly, once we have identified a candidate microbe, therapeutic interventions are often designed to modify its presence or activity where health and disease most obviously manifest: within an individual host. This playbook for investigating host-microbe interactions has been successful and will undoubtedly continue to guide us. Indeed, specific microbial strains or lineages have been linked to microbiome activities such as educating the immune system (8), inhibiting pathogen colonization (2), and sparking inflammation (9). However, the default “one host, one microbe” view of host-microbe interactions may be restricting our field of vision. An expanded and more integrated view would superimpose our current working definitions of host-microbe interactions onto a broader ecological and systems-level framework that encompasses interacting networks of hosts, the environment, and the microbes that connect them. In this wider panorama, new and alternative therapeutic interventions come into focus. Rather than treating afflictions at the scale of a single host and microbe, interventions could be designed to canalize entire host-microbe systems in a way that channels health-promoting microbial factors or processes while diverting those that drive disease.

## THE THERAPEUTIC POTENTIAL OF MICROBIOME CONNECTIVITY

Our personal microbiomes are often referred to as a “microbial organ” to emphasize the vital functions they provide us. Although this comparison is largely taken to be metaphorical, it does convey some truth. As with actual organs such as the heart or liver, microbiomes can be transplanted from one host to another. Experimentally, this attribute has been exploited to demonstrate the sufficiency of microbiome-encoded activities to modify or confer certain host phenotypes (e.g., inflammation [10]). Clinically, fecal microbiota transplants have been used in humans to repair damaged microbiomes after antibiotic treatment (11) and improve responsiveness to cancer immunotherapy (12). But, outside controlled experimental and clinical applications, a property of microbiomes that is not shared with real organs is that microbiome transmissibility is a natural and ongoing process (13, 14).

Signatures of microbial flux among humans, animals, and the environment continue to be detected by increasingly large-scale DNA sequencing-based microbiome projects. For example, taxa from a mother’s microbiome can be vertically transferred to her newborn child during birth (15), and there is evidence of microbiota transmission among cohabiting family members, housemates, and pets (16, 17). There are microbial exchanges between people and the built environment as well, which in some cases can occur on surprisingly rapid time scales. For example, hospital patients can start acquiring microbes from the rooms they occupy within the first 24 h of their stay (18). Likewise, hospital rooms accumulate microbial traces of the patients they house over time. But perhaps some of the most striking signatures of microbiome transmission come from studies charting the spread of antibiotic-resistant bacteria.

Antibiotic-resistant bacteria are an intensifying public health threat. The urgency of this problem has prompted the tracking of antibiotic-resistant bacteria and the resistance genes they carry throughout the biosphere and across human and animal populations. Monitoring the movements of antibiotic-resistant bacteria is essential for preventing incurable infections and informing newly emerging “One Health” policies. However, an added benefit of this surveillance is that it helps define the possible routes and dynamics of microbial transmission that connect microbiomes.

Drug-resistant bacteria and resistance genes are alarmingly omnipresent. Reflecting this fact, a recent study of mass transit systems from 60 cities across the world revealed that antibiotic resistance genes are common in urban microbiomes (i.e., the microbial communities that live on the surfaces of urban built environments) (19). Notably, urban microbiomes appear to contain city-specific resistance gene fingerprints, highlighting a potential for globe-trotting humans to acquire and spread antibiotic-resistant bacteria. Demonstrating this potential, multiple studies have indicated that, on average, one-third of international travelers may acquire bacteria carrying genes that encode extended-spectrum beta-lactamases or resistance to last-resort antibiotics such as colistin and carbapenems (20, 21). In some regions of Asia and Africa acquisition rates were observed to be near or above 50%. It was further observed that travel-acquired drug-resistant bacteria could reside within an individual traveler’s microbiome for up to a year after returning home and, in some instances, could also be transmitted to another household member (20).

On a regional scale, the epidemiology of plasmid-based colistin resistance (specifically, *mcr-1*) signals the potential for transmission of drug-resistant bacteria through production animal supply chains. Unexpectedly high rates of *mcr-1* carriage (15% on average) were recently detected in fecal samples of healthy human individuals across numerous provinces and municipalities in China (22). One of the strongest predictors of *mcr-1* carriage is a high daily intake of aquatic food products. This link is notable because it has been proposed that agricultural runoff contaminated with colistin may have first stimulated the emergence and amplification of *mcr* genes in aquatic bacteria (23–25). In turn, it is thought that integrated aquaculture practices and consumption of aquaculture-derived food have led to the horizontal transfer of *mcr* genes to terrestrial and human-associated bacteria (24, 25).

**FIG 1:**
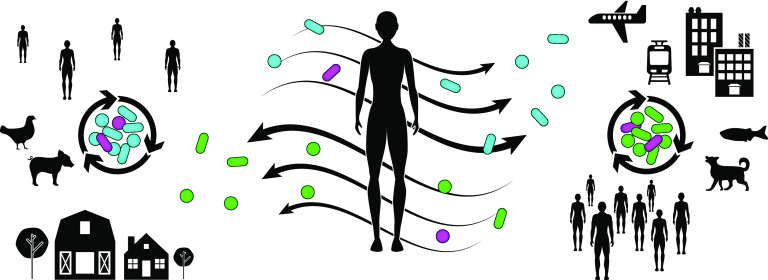
A picture of microbial flow that connects microbiomes. Depicted on the left and right are two biogeographically distinct regions that are defined by different human population densities, travel patterns, domesticated animals and food supply chains, and built environments (rural versus urban). Each region harbors unique microbial assemblages (cyan versus green microbes). Magenta microbes represent shared taxonomic lineages that display divergent traits or behaviors (e.g., microbes carrying mobile genetic elements containing antibiotic resistance genes). The central figure represents an individual human who both contributes to and is affected by microbial transmission within and between microbiomes.

Together, the examples above begin to outline a picture of microbial flow that connects human, animal, and environmental microbiomes (Fig. 1). With this picture in mind, it becomes difficult to ignore the potential for continuous microbial transmission to shape and reshape our microbiomes—which in turn could shape and reshape our biology. I specifically called attention to antibiotic resistance as a pronounced example of how microbial transmission and microbiome connectivity can underlie public and individual health. But similar processes are likely at work for a range of microbiome-based diseases. For example, it is possible that chronic diseases that are typically thought of as noncommunicable and labeled as epidemic may be driven, in part, by actual epidemics of microbial spread. Conversely, it is tempting to speculate whether outbreaks and epidemics of health involving the widespread transmission of beneficial microbes are possible. In my laboratory, these ideas are motivating research projects aimed at piecing together a broader, mechanistic view of the microbial life cycles that shape and connect microbiomes. We are seeking answers to questions like: Where do the individual microbes that comprise microbiomes come from? What controls whether they colonize and assemble into a community or disperse to new hosts and throughout the environment? How do the life history and evolution of microbes impact the form and function of microbiomes? Adding to the allure of exploring these questions is the prospect of resolving the interconnections among the multitude of nested life cycles within microbiomes—spanning hosts, bacteria, viruses, and mobile genetic elements. Ultimately, I think attaining a systems-level view of host-microbe interactions that captures the mechanisms driving microbial life cycles will inspire new therapeutic interventions that promote individual health by targeting and controlling microbiome connectivity.
